# Household

**Published:** 1887-03

**Authors:** 


### HOUSEHOLD.

GRAHAM MUSH is a good substitute for a rich pudding on some occasions. Make just as you do corn meal mush, but add a few berries or raisins, or English currants. Serve with milk and sugar.

ONE EGG CAKE—Rub two large spoonfuls of butter into a heaping cup of white sugar. Add a beaten egg and a cup of sweet milk, with one teaspoonful of soda dissolved in it. Mix two teaspoonfuls of cream tartar into two heaping cups of flour, and stir in. Flavor to taste. Bake in deep pans.

BAKED MACARONI—Wash the macaroni, and put it in a sauce-pan, with sufficient water to cover it; boil one half hour; put alternately in a pudding-dish, a layer of boiled macaroni and grated cheese, seasoning each layer of macaroni with salt and pepper; have the top layer of cheese, with a teaspoonful of butter in the center; pour over it one-half pint of sweet milk; bake one half hour.

STEWED APPLES.—Peel and slice six good-sized apples; put in a stew-pan a pint of water, and one-half pint of white sugar; when this boils, drop in the apples and two or three slices of lemon. Boil until the apples are clear, but not until they go to pieces. Serve cold, with cream or without.

FRIED SCALLOPS.—Wipe each roll in beaten egg, then in fine crumbs, and fry in hot lard or dripping to a fine brown. Shake off the fat in a split spoon and lay in rows on a hot dish. Garnish with parsley. Pass hot crackers, mashed potato and cut lemon with them.

APPLE BATTER PUDDING.—Put into a bowl half a pound of flour, add a pinch of salt, and stir in very gradually half a pint of new milk. Beat it until smooth, then add three eggs. Pour about half the mixture into a buttered pie dish, and put it into the oven to get firm. Then nearly fill the dish with apples pared, cored, sliced and slightly stewed with a little sugar and lemon rind. Pour the rest of the batter over them, return to the oven and bake one hour and a half.

CHEERFUL HOMES.—See to it, that your home competes with public places in attractiveness. Open your blinds by day and light bright fires at night. Illuminate your homes. Hang pictures upon the walls. Put books and newspapers on your tables. Have music and entertaining games. Banish demons and dullness and apathy, that have so long ruled in your household, and bring in mirth and good cheer. Invent occupations for your sons. Stimulate their ambitions in worthy directions. While you make home their delight, fill them with higher purposes than mere pleasure. Whether they should pass happy boyhood and enter upon manhood with refined tastes and noble ambitions depends on you. Believe it possible that with exertion and right means a mother may have more control over the destiny of her boys than any other influence whatever.—*Omaha Daily Republican.*

The Laundry.—A spoonful of oxgall to a gallon of water will set the colors of almost any goods soaked in it previous to washing. A teacup of lye in a pail of water will improve the color of black goods. Napkins should lie in lye before being washed ; it sets the color. A strong tea of common hay will preserve the color of French linen. Vinegar in the rinsing water for the pink or green calicoes will brighten them : soda answers the same end for both purple and blue. To bleach cotton cloth, take one large spoonful of sal-soda and one pound of chloride of lime for thirty yards ; dissolve in clean soft water ; rinse the cloth thoroughly in cold soft water so that it may not rot. This amount of cloth may be bleached in fourteen or fifteen minutes.

Boiled eggs which adhere to the shell are fresh. A good egg will sink in water. Stale eggs are glassy and smooth of shell. A fresh egg has a lime-like surface to its shell. After an egg has been laid a day or more the shell comes off easily when boiled. A boiled egg which is done and will dry quickly on the shell when taken from the kettle is fresh. Eggs which have been packed in lime look stained and show the action of the lime on the surface. If packed in bran for a long time eggs are apt to acquire a musty smell and taste.

Young Girls Learning to Cook.—Over one hundred girls, whose ages range from eleven to fifteen years, their faces wreathed in smiles and their heads crowned with neat little cooking-caps, were gathered together recently at No. 28 Lafayette Place. They were pupils of the public schools and had met to show what they had learned during the last summer vacation, in the New York Cooking School. The exercises consisted of songs, recitations and answering of questions in concert, all bearing on cookery or on some article of food. Morris K. Jesup read the annual report of the school, showing that the institution is in a prosperous condition.

At the close of the exercises there was an exhibition of various dishes prepared by the pupils without any assistance from their teachers, and roasted fowls, delicious puddings, pies and cakes, jellies, bread, salads and other viands were displayed in a tempting array.

To Make Apple Float.—To each apple, after it is stewed and run through a sieve, put three large tablespoonfuls of sugar and the white of one egg, beaten to a stiff froth. Beat all together with a fork, and flavor with lemon. Allow it to cool.

A Nice Breakfast Dish can be made of scraps of boiled ham. Chop them fine, and heat by throwing them into a hot frying pan. Beat two or more eggs, according to the quality of ham and pour into a hot, buttered pan on the stove ; when it is brown on one side, spread the ham on half of it, and turn the other half over it.

